# Individual heterogeneity in ixodid tick infestation and prevalence of *Borrelia burgdorferi* sensu lato in a northern community of small mammalian hosts

**DOI:** 10.1007/s00442-023-05476-w

**Published:** 2023-11-13

**Authors:** Lars K. Lindsø, Jason L. Anders, Hildegunn Viljugrein, Anders Herland, Vetle M. Stigum, W. Ryan Easterday, Atle Mysterud

**Affiliations:** 1 Centre for Ecological and Evolutionary Synthesis (CEES), Department of Biosciences, University of Oslo, Blindern, P.O. Box 1066, NO-0316 Oslo, Norway; 2 Norwegian Veterinary Institute, P.O. Box 64, NO-1431 Ås, Norway; 3 Norwegian Institute for Nature Research (NINA), Torgarden, P.O. Box 5685, NO-7485 Trondheim, Norway

**Keywords:** Ixodes ricinus, Tick aggregation, Tick-borne pathogens, Lyme disease, Sex bias

## Abstract

Heterogeneous aggregation of parasites between individual hosts is common and regarded as an important factor in understanding transmission dynamics of vector-borne diseases. Lyme disease is vectored by generalist tick species, yet we have a limited understanding of how individual heterogeneities within small mammal host populations affect the aggregation of ticks and likelihood of infection. Male hosts often have higher parasite and infection levels than females, but whether this is linked to sexual body size dimorphism remains uncertain. Here, we analysed how host species, sex, and body mass influenced *Ixodes ricinus* tick infestations and the infection prevalence of *Borrelia burgdorferi* sensu lato (s.l.) in three species of small mammals involved in the enzootic transmission cycle of Lyme disease in Norway from 2018 to 2022. Larval and nymphal ticks were found on 98% and 34% of all individual hosts, respectively. In bank voles and wood mice, both larval and nymphal tick infestation and infection probability increased with body mass, and it increased more with mass for males than for females. Tick infestation in the common shrew increased with body mass and was higher in males, while pathogen infection was higher in females. Sex-biases in infestation did not correspond with level of sexual body mass dimorphism across species. This study contributes to our understanding of how individual heterogeneity among small mammalian hosts influences *I. ricinus* tick aggregation and prevalence of *B. burgdorferi* s.l. at northern latitudes.

**Supplementary Information:**

The online version contains supplementary material available at 10.1007/s00442-023-05476-w.

## Introduction

Parasites are often heterogeneously distributed among individuals in host populations whereby some individuals have higher parasite load compared to others (Shaw et al. 1998). Individual heterogeneity in parasitism is often linked to differences in host traits that affect either exposure or susceptibility to parasites (Wilson et al. 2002; Brunner and Ostfeld 2008; Guerra-Silveira and Abad-Franch 2013). One commonly observed pattern is that males often are more parasitised than females (Córdoba-Aguilar and Munguía-Steyer 2013; Metcalf and Graham 2018). However, demographic patterns of parasitism are not always consistent across (Kiffner et al. 2013; Smyth and Drea 2016) or within host taxa (Cull et al. 2017). The propensity of ectoparasites to aggregate on certain individuals is an important question due to its implications for the dynamics of vector-borne pathogens and how their transmission might be focussed on certain groups in the host population (Brunner and Ostfeld 2008; Kilpatrick et al. 2017).

Ticks within the Ixodidae family are important vectors of several zoonotic pathogens that cause disease in humans in the Northern Hemisphere (Piesman and Gern 2004), including Lyme disease caused by specific genospecies within the *Borrelia burgdorferi* sensu lato (s.l.) complex (Franke et al. 2013). The generalist sheep tick, *Ixodes ricinus*, is the predominant vector of these pathogens in Europe (Estrada-Peña and Jongejan 1999) and is currently shifting its distribution range to higher latitudes and elevation due to climate warming and land use change (Medlock et al. 2013; Swei et al. 2020). *Ixodes ricinus* larvae hatch from their eggs and require a blood meal from a vertebrate host to moult into nymphs that subsequently need another blood meal the following year to moult into adults (van Duijvendijk et al. 2016). Evidence suggests that hatched *I. ricinus* larvae are rarely infected by *B. burgdorferi* s.l. (Richter et al. 2012; Rollend et al. 2013). Rather, larvae become infected by feeding on an infected host or by co-feeding in the vicinity of an infected nymph on the same host (Gern and Rais 1996; Voordouw 2015). Identifying the factors that influence tick abundance and aggregation is, therefore, crucial for understanding the contribution of different host groups to pathogen transmission (Keesing et al. 2010; Mccoy et al. 2013; Kilpatrick et al. 2017).

The most common pathogenic genospecies causing Lyme disease in Europe, *Borrelia afzelii*, has a natural reservoir in small mammals (Gern et al. 1998; Hofmeester et al. 2016). While *I. ricinus* feeds on a wide range of vertebrate hosts, small mammals are considered a particularly important group due to their role in feeding a large proportion of larvae (Paziewska et al. 2010; Bown et al. 2011; Mysterud et al. 2015; van Duijvendijk et al. 2016). Small mammals are also reservoir hosts for several tick-borne pathogens (Gern et al. 1998; Hersh et al. 2014; Obiegala et al. 2014; Hofmeester et al. 2016; Cayol et al. 2017). Previous studies at the northern distribution range of *I. ricinus* have focussed on identifying key host species for the epidemiological cycle of Lyme disease including small mammals with potential reservoir competence for *B. burgdorferi* s.l. (Mysterud et al. 2019b; Sormunen et al. 2023) and relative tick aggregation across species (Mysterud et al. 2015, 2019b; De Pelsmaeker et al. 2022). However, ticks may also aggregate on specific individuals within host populations resulting in differential patterns of infestation (i.e. number of ectoparasites) and infection prevalence among individual hosts (Brunner and Ostfeld 2008; Kilpatrick et al. 2017).

In small mammalian hosts, body mass has been identified as an important factor for tick aggregation, whereby larger hosts tend to harbour more ticks, especially nymphs (Perkins et al. 2003; Mysterud et al. 2015). In voles and mice, studies have reported proportionally higher tick burdens in males compared to females (Tälleklint and Jaenson 1997; Harrison et al. 2010; Dallas et al. 2012; Perez et al. 2017). Harrison et al. (2010) suggested that the observed sex-bias in tick burden is due to sexual dimorphism in body mass. On the other hand, other studies have suggested a testosterone mediated trade-off between immune defence and reproduction in male bank voles (Hughes and Randolph 2001) and wood mice (Hughes and Randolph 2001; Mills et al. 2010), whereby males invest more into reproduction at the expense of parasite defence, resulting in higher tick burdens in sexually active males. At the northern distribution range, there is a limited understanding of aggregation patterns of *I. ricinus* in host communities with multiple small mammal species. This includes less studied host species like the common shrew *Sorex araneus* (Bown et al. 2011; De Pelsmaeker et al. 2022; Sormunen et al. 2023), and whether patterns of tick aggregation affect the transmission dynamics of *B. burgdorferi* s.l. Shrews have lower levels of sexual body-size dimorphism compared to rodents, and this could allow for testing of mechanisms causing sex-biased parasitism.

In the present study, we analysed the infestations of *I. ricinus* ticks and prevalence of *B. burgdorferi* s.l. in 415 individuals from a community of small mammalian host species over 5 years (2018–2022) in a boreonemoral forest in South-East Norway. The small mammal community consisted of five species, including the wood mouse (*Apodemus sylvaticus*), field vole (*Microtus agrestis*), bank vole (*Myodes glareolus*), common shrew (*Sorex araneus*), and pygmy shrew (*Sorex minutus*). We analysed how species, body mass, sex, and their interactions affected larval and nymphal tick infestations and infection prevalence of *B. burgdorferi* s.l. in hosts. We hypothesised that: (I) A) tick infestations and B) pathogen infection prevalence was positively associated with body mass; (II) A) tick infestations and B) pathogen infection prevalence to be higher in males than females; (III) level of sex-biased tick infestation differed between species of small mammals dependent upon sexual body-size dimorphism.

## Materials and methods

### Study area

**Fig. 1:**
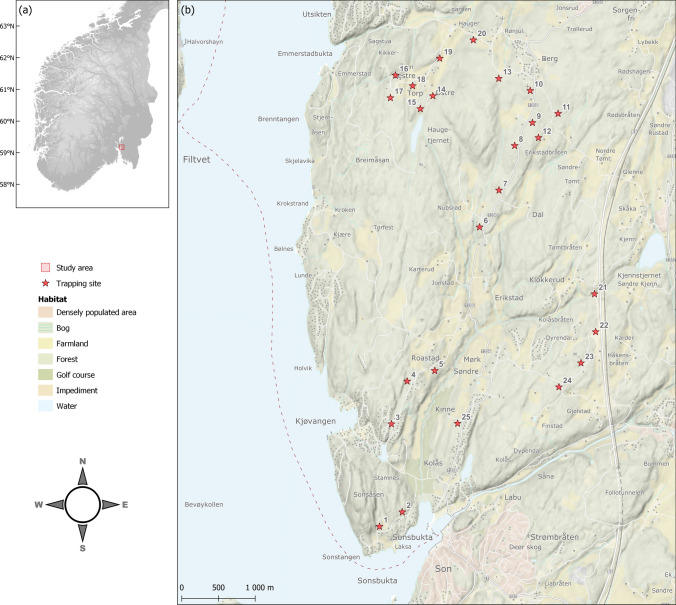
Map of **a** the main location of the study area in Norway and **b** the spatial distribution of 25 small mammal trapping sites

The study area is located in Vestby, Viken in South-East Norway (Fig. 1). The area represents a habitat mosaic of smaller settlements, farmland (mainly growing grain), and managed forests. It is part of the boreonemoral zone (Abrahamsen et al. 1977) with an average annual temperature of 3.4 °C and average total precipitation of 735 mm (Norwegian meteorological station no. 03780, met.no). Forests are mostly coniferous, dominated by Scots pine (*Pinus sylvestris*) and Norway spruce (*Picea abies*) with an interspersion of birch (*Betula* spp.). Some areas with richer soils and favourable local climate are dominated by deciduous trees, predominantly oak (*Quercus* spp.) and Scots elm (*Ulmus glabra*). The field layer was typically composed of bilberry (*Vaccinium myrtillus*), heather (*Calluna vulgaris*), peat moss (*Sphagnum* spp.), and various forbs and graminids (Gramineae). Roe deer (*Capreolus capreolus*) are abundant and the main hosts for adult *I. ricinus* ticks in this ecosystem (Mysterud et al. 2021).

### Trapping

We captured small mammals in the spring (i.e. late May) and autumn (i.e. late August) from 2018 to 2022. We had 25 trapping sites (Fig. 1) with a minimum distance of 500 m from one site to another to avoid local depletion of populations (Mysterud et al. 2019b). All trapping sites were proximate to roads for logistical reasons, but with a minimum distance of 50 m. Four trap stations were placed in a square formation at each trapping site in accordance with the small quadrate method (Myllymäki et al. 1971). We used a combination of common snap traps and live cage traps (Grahnab AB Ugglan Special No. 3). These two trap types had no measurable impact on tick infestation in a study on this specific topic (De Pelsmaeker et al. 2020), and we found no measurable effects of whether the animal was found alive or dead in a supporting analysis detailed in the appendices (Table S6). Each of the four trap stations at each trapping site consisted of either 1 cage trap or 3 snap traps (to avoid trap saturation) in a triangle formation. Within 2 m of each trap site corner, each trap station was positioned in the terrain to maximise the probability of capture. The trap coordinates and elevations were recorded using a handheld GPS. The traps were set and baited the first day and checked once in the morning for three consecutive days throughout the trapping period. All traps were baited with oats and raisins. The cage traps were covered with a metal sheet to protect trapped animals from predators and weather, and a piece of carrot was placed inside for sustenance. Cage-trapped animals were euthanised by cervical dislocation. All trapped animals were kept in individual plastic bags with an individual ID, trap number, and date of capture, and stored in a – 20 °C freezer for later examination in the laboratory. All samples were processed within 2 years of trapping.

### Physical laboratory examinations

All animals were weighed and identified to species. Species determination was based on morphological characteristics and performed by an expert on small mammals (Jeroen van der Kooij). Sexing of rodents was done by dissection and identification of either the ovaries in females or testes in males. Sexing of shrews was done following an established PCR assay and protocol for shrews (Matsubara et al. 2001). Each individual animal was examined for ticks using a magnifying glass and tweezers. A standardised examination time of minimum 20 min was set to ensure that all individuals were given equal examinations following previous studies (Mysterud et al. 2015, 2019b). During the examination, all ticks were placed aside before being counted. Lastly, the developmental stage (larvae, nymph, or adult) was determined under a stereomicroscope. For ticks from 2020 to 2022, we also determined each tick to species based on morphological characteristics (Fig. S1) (Arthur 1963), while we used genetic determination for a subset from 2018 to 2019 (see below). After tick examination, a tissue sample from each animal’s ear was collected, stored in 96% ethanol in a – 20 °C freezer to be used in pathogen detection.

### Genetic analyses

We used an established real-time multiplex quantitative PCR (qPCR) protocol (Courtney et al. 2004) implemented in the laboratory at the Centre for Ecological and Evolutionary Synthesis (CEES) at the University of Oslo to detect the presence of *B. burgdorferi* s.l. in ear tissue of small mammals (Mysterud et al. 2013, 2016, 2019a, b). We previously showed that all *B. burgdorferi* s.l. sequences from small mammals in our study area come from the genospecies *B. afzelii* (Mysterud et al. 2019a). The qPCRs were carried out on a Roche Lightcycler® 96 instrument. The qPCR was performed in 10 μl reactions composed of 2.0 μl of 5 × PCR Buffer (F. Hoffmann-La Roche Ltd.), 5.25 μL of PCR-grade H_2_O, 0.7 μl of 0.7 µM primer mix and 0.0875 μl of 0.175 µM probes for *B. burgdorferi* s.l. (Table S1), and 1 μl of DNA template. The two last samples of each 96-well plate were controls: one positive and one negative (non-template control). Quality checked DNA samples analysed in a preliminary qPCR analysis that were infected with *B. burgdorferi* s.l. at cycle threshold (CT) values < 25 were pooled and used as positive controls, while 1 μL of RNase-free H_2_O was used as negative controls. The PCR conditions were 95 °C for 10 min of preincubation, followed by 2-step amplification of 95 °C for 15 s and 60 °C for 60 s for 50 cycles. We analysed the qPCR results and assigned infection status of each sample in the application LightCycler® 96 version 1.1.9.1320.

To verify morphological species determination of ticks and to identify a subset of ticks (2018–2019) to species, we used a novel multiplex qPCR assay for identification of *I. ricinus* and *I. trianguliceps* (unpubl.) on a subset of morphologically determined species of ticks (*n* = 32) using two replicates of DNA template (unpubl. results). The qPCR was performed in 10 μl reactions with 2.0 μl of 5 × PCR Buffer (F. Hoffmann-La Roche Ltd.), 5.6 μL of PCR-grade H_2_O, 0.6 μl of 0.6 µM primer mix and 0.1 μl of 0.2 µM probes for *I. ricinus*, and 0.6 μl of 0.6 µM primer mix and 0.1 μl of 0.2 µM probes for *I. trianguliceps* (Table S1), and 1 μl of DNA template. The two last samples of each 96-well plate were positive and negative controls, respectively. We pooled confirmed *I. ricinus* and *I. trianguliceps* samples as positive controls and used 1 μL of RNase-free H_2_O as negative controls. The PCR conditions were the same as those for determining *B. burgdorferi* s.l. infection status.

### Statistical analyses

Statistical analyses were conducted in R version 4.2.1 (R Core Team 2022). Due to low sample size of two species, all analyses were restricted to the three most abundant host species found in the study area (i.e. bank vole, common shrew, and wood mouse). To investigate how body mass varied between seasons and sexes in the different species, we analysed body mass as the response variable in generalised linear mixed models (GLMMs) with a Gamma distribution (log-link) to account for the skewed distribution of body mass. To account for the possible influence of pregnant females on sexual body mass dimorphism, we excluded all pregnant females in this analysis. We built a global model that included host species, sex, and season as factor variables, all possible interaction terms between the factor variables, and trap station and year as random intercepts.

**Fig. 2:**
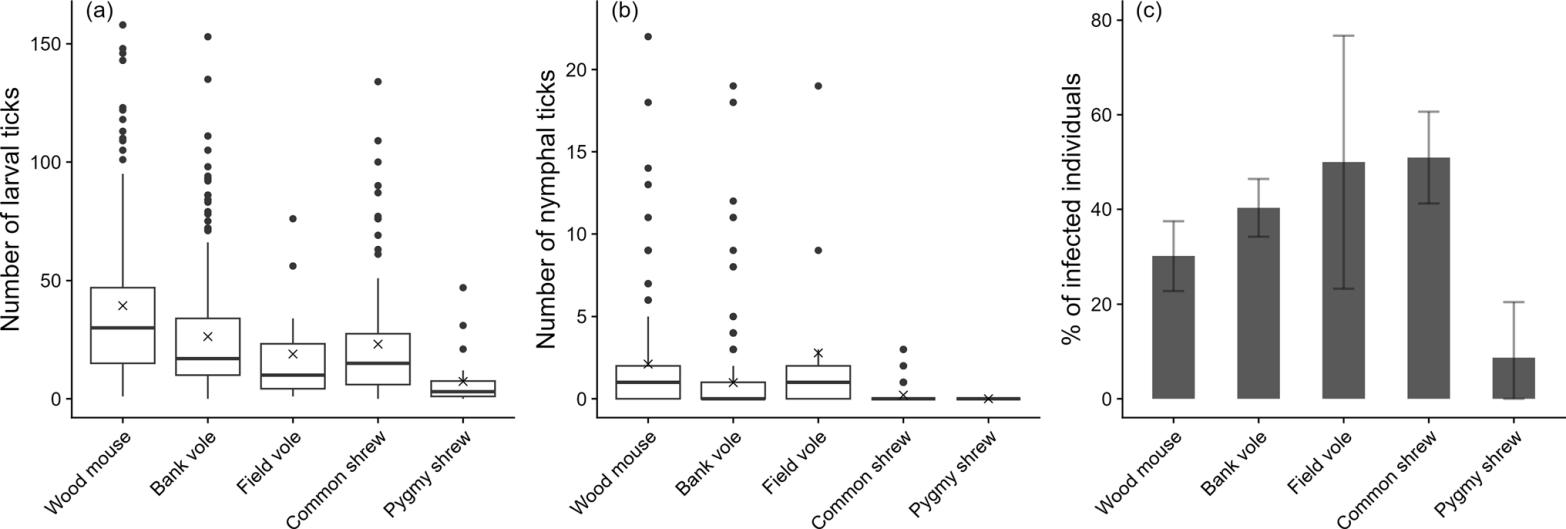
Boxplot of **a** larval and **b** nymphal ixodid tick infestations and **c** average prevalence of *Borrelia burgdorferi* s.l. in captured wood mice (*Apodemus sylvaticus*), bank voles (*Myodes glareolus*), field voles (*Microtus agrestis*), common shrews (*Sorex* *araneus*), and pygmy shrews (*Sorex minutus*) in South-East Norway (2018–2022). The × in a and b denote respective mean values and error bars in c denote respective 95% binomial proportion confidence intervals

We calculated the proportion of individuals with larval and nymphal ticks and *B. burgdorferi* s.l. infections for each species, and their respective 95% binomial proportion confidence intervals by adding and subtracting 2 standard errors. Species identification of ~ 13,000 ticks revealed a low proportion of the specialist tick *I. trianguliceps* larvae (~ 1%) and nymphs (< 4%; Table S3; Table S4), and both total larval and nymphal tick counts were highly correlated with the number of *I. ricinus* larvae (Pearson correlation *r* ≈ 1) and nymphs (*r* ≈ 0.99), respectively. Therefore, we analysed parasite burdens across both species of ticks using GLMMs. We built a global model that included host species, sex, and season as factor variables and log-transformed body mass, centred per species to account for different distributions of body mass among species, as a continuous variable. We also included interaction terms for species, sex, and species-centred (log) body mass. For each species, females of higher body mass than the male maximum were truncated down to the male maximum body mass in order to reduce the impact of outliers. Trapping station and year were included as random intercepts. Larval tick intensities were analysed with negative binomial GLMMs fitted jointly for all three species. Due to a low presence of ixodid nymphs on hosts, nymphal tick presence (or absence) was analysed across all three species with mixed effects logistic regression models. In addition, all hosts with nymphs were analysed in a supplementary analysis to investigate effects on nymphal tick intensity, i.e. the number of nymphs among hosts with nymphs (Margolis et al. 1982), using zero-truncated negative-binomial GLMMs. Shrews were excluded from this analysis due to a low presence of nymphs (Fig. 2b).

We analysed the prevalence of *B. burgdorferi* s.l. with mixed effects logistic regression models. We built a global model that included host species, sex, and season as factor variables, and species-centred log-transformed body mass as a continuous variable. Interaction terms for species, sex, and species-centred (log) body mass were also included, and trapping station and year were included as random intercepts. In addition, we ran a model for which shrews were again excluded to test whether nymphal tick presence influenced pathogen prevalence.

All models were fitted using the R package glmmTMB version 1.1.4 (Brooks et al. 2017). Model selection was done using backward selection procedure with Akaike Information Criterion (AIC) in the package MuMIn version 1.47.1 (Barton 2022) for both the tick models and the pathogen model. For each analysis, we retained only the simplest of the top models within a ΔAIC score of 2.0 (Burnham and Anderson 1998). Residuals were inspected for potential remaining patterns with the available parameters. If the residuals of the top model indicated remaining non-linear patterns of body mass, we included a second-order polynomial for species-centred (log) body mass. Patterns of tick infestation, infection, species-centred (log) body mass, and sex in the models used for inference were inspected for consistency in separate tick and infection models run for species- and season-specific subsets of the data. Finally, to test for potential confounding effects of season and body mass, we fitted separate models with identical parameters to the parsimonious models but using season as a substitute for body mass. Interactions were not included.

## Results

In total, we trapped 557 individuals of 5 small mammal species in 2018–2022. The most abundant small mammal species were the bank vole (*n* = 258), wood mouse (156), and common shrew (106), while the pygmy shrew (23) and field vole (14) were less common (Table S2). The sex ratio (males per total catch) was 0.56 in wood mice, 0.43 in the field vole, 0.52 in the bank vole, 0.51 in the common shrew, and 0.39 in the pygmy shrew.

The 557 captured hosts harboured 16,452 ticks in total, of which 13,137 were identified to species by qPCR and morphology (Table S3). The presence of larval ticks was very high (96–100% [95% CI 93–100]) in all host species but less so for the pygmy shrew (78% [60–97]). The presence of nymphal ticks was highest on the field vole (57% [28–87]), followed by the wood mouse (51% [43–59]), bank vole (33% [27–39]), and common shrew (15% [8–22]). No nymphs were found on pygmy shrews. Species identification of a subset of morphologically identified *I. ricinus* (*n* = 18) and *I. trianguliceps* (*n* = 14) was consistent across qPCR results with two replicates, and species proportions were similar between ticks identified with qPCR and by morphological traits (Table S3). Of 12,548 larval ticks identified to species, 12,429 (99%) were *I. ricinus*. Of 589 nymphal ticks identified to species, 561 (95%) were *I. ricinus*. We, therefore, refer to tick load as by *I. ricinus*. A detailed overview of larval and nymphal tick infestation for all the host species is given in the Appendices (Table S4). Common shrews were most often infected with *B. burgdorferi* s.l. (51% [41–61]), followed by field voles (50% [21–79]), bank voles (41% [34–46]), wood mice (30% [23–37]), and pygmy shrews (9% [0–21]; Fig. 2c).

**Fig. 3:**
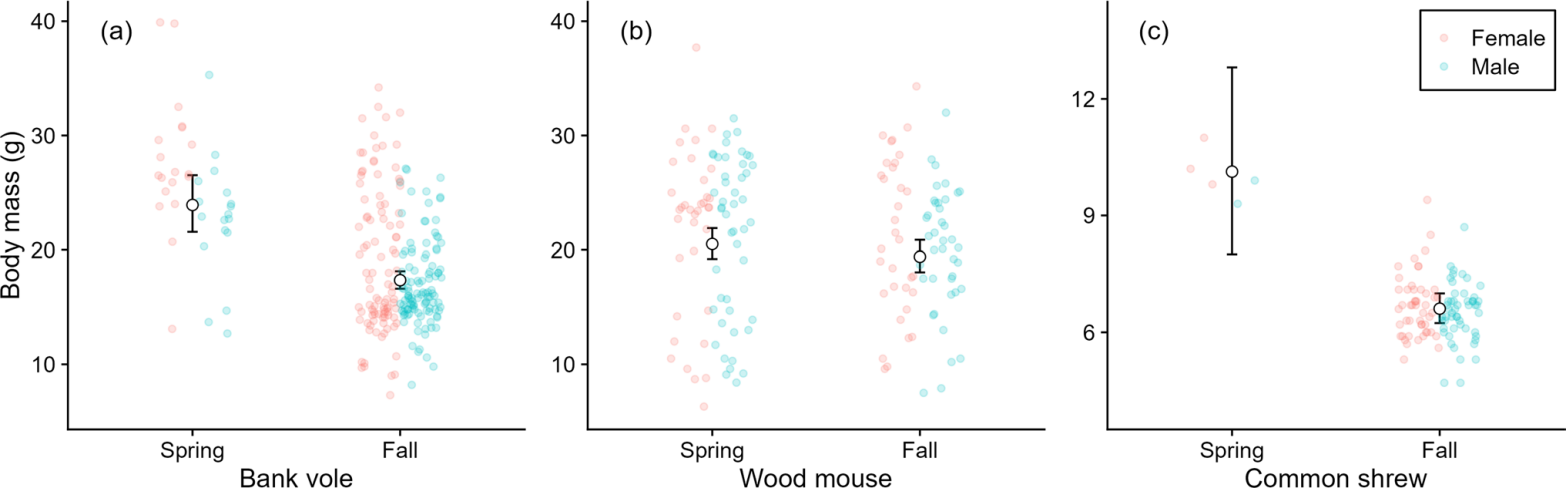
Predicted body mass by season in **a** bank voles, **b** wood mice, and **c** common shrews captured in South-East Norway (2018–2022). Error bars denote respective 95% confidence intervals and jittered points denote all raw data observations, coloured by sex

The average body mass was 21 g (range 6.3–38) in the wood mouse, 19 g (7.3–40) in the bank vole, and 6.8 g (4.7–11) in the common shrew. The best supported model of body mass included species and season, in addition to the interaction between “species × season”, but not “sex” and the interaction between “species × sex” and “season × sex” (Table S5). Wood mice were heavier than bank voles, and common shrews were significantly smaller than bank voles (Table 1). There were no significant sex differences. All species were heavier in spring than fall (1.4 times [95% CI 1.2–1.5] higher for bank voles, 1.7 [1.3–2.2] times higher for common shrews, and 1.1 [0.9–1.2] times higher for wood mice), but not significantly for the wood mouse (Fig. 3; Table 1).

**Table 1:** Parameter estimates from the best models on body mass, tick infestation, and infection in captured bank voles, wood mice, and common shrews in South-East Norway (2018–2022)

Parameter	Estimate	Std. error	*z*	*P*
Body mass (all species)	(log-link)			
Intercept^a^	2.854	0.022	127.560	< 0.001
spCommon shrew	– 0.966	0.033	– 29.220	< 0.001
spWood mouse	0.111	0.040	2.750	0.006
SeasonSpring	0.320	0.055	5.850	< 0.001
spCommon shrew: seasonSpring	0.107	0.134	0.790	0.427
spWood mouse: seasonSpring	– 0.265	0.072	– 3.680	< 0.001
Larval tick infestation (all species)	(log-link)			
Intercept^b^	3.186	0.164	19.478	< 0.001
spCommon shrew	– 0.416	0.092	– 4.513	< 0.001
spWood mouse	0.294	0.079	3.727	< 0.001
Centred (log) mass	0.625	0.142	4.408	< 0.001
SexMale	0.255	0.064	3.955	< 0.001
Centred (log) mass: sexMale	0.800	0.217	3.686	< 0.001
Nymphal tick presence (all species)	(logit-link)			
Intercept^b^	– 0.667	0.290	– 2.301	0.021
spCommon shrew	– 1.321	0.342	– 3.864	< 0.001
spWood mouse	0.562	0.246	2.286	0.022
Centred (log) mass	1.720	0.486	3.536	< 0.001
SexMale	0.505	0.221	2.290	0.022
Centred (log) mass: sexMale	2.322	0.814	2.853	0.004
Nymphal tick intensity (rodents)	(log-link)			
Intercept^c^	0.066	0.303	0.217	0.828
Centred (log) mass	0.992	0.654	1.518	0.129
SexMale	0.326	0.278	1.171	0.241
Centred (log) mass: sexMale	2.501	0.914	2.735	0.006
Infection prevalence 1 (all species)	(logit-link)			
Intercept^b^	0.376	0.326	1.155	0.248
spCommon shrew	– 0.269	0.295	– 0.910	0.363
spWood mouse	– 1.373	0.307	– 4.470	< 0.001
SexMale	0.162	0.229	0.708	0.479
Centred (log) mass	0.236	0.042	5.566	< 0.001
Centred (log) mass^2^	– 0.017	0.004	– 4.126	< 0.001
SexMale: centred (log) mass	0.132	0.055	2.398	0.017
spCommon shrew: centred (log) mass	– 7.444	1.762	– 4.225	< 0.001
spWood mouse: centred (log) mass	0.859	1.551	0.554	0.580
spCommon shrew: sexMale	– 1.412	0.560	– 2.519	0.012
spWood mouse: sexMale	– 0.141	0.616	– 0.229	0.819
Infection prevalence 2 (rodents)	(logit-link)			
Intercept^d^	– 0.739	0.315	– 2.348	0.019
spWood mouse	– 1.392	0.304	– 4.573	< 0.001
Sexmale	0.525	0.277	1.899	0.058
Centred (log) mass	3.107	0.644	4.825	0.000
Nymph presence	0.867	0.277	3.132	0.002
Centred (log) mass: sexMale	2.836	1.115	2.544	0.011

^a^Corresponds to bank vole in fall

^b^Corresponds to mean body mass and female bank vole

^c^Corresponds to mean body mass and female

^d^Corresponds to mean body mass and female bank vole with no nymphs

**Fig. 4:**
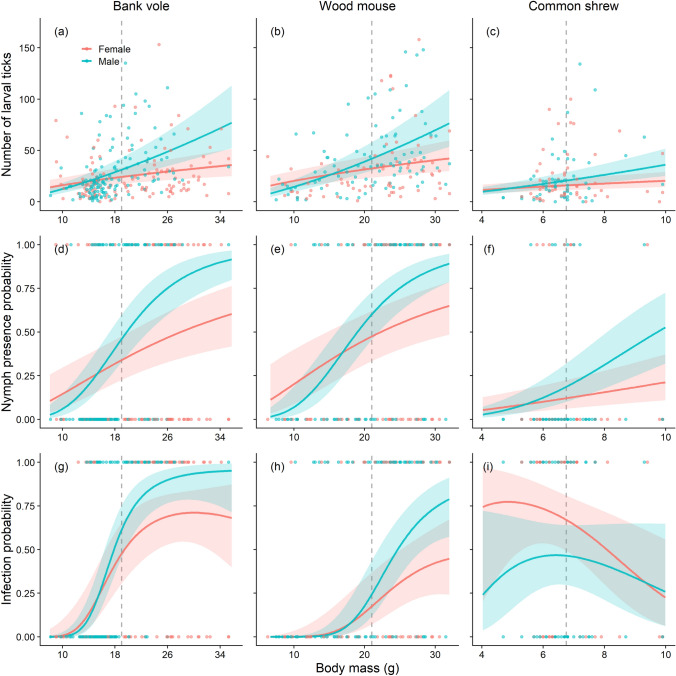
Predicted number of *Ixodes* larvae (**a–c**), presence of *Ixodes* nymphs (**d–f**), and prevalence of *Borrelia burgdorferi* s.l. (**g–i**) as a function of sex and body mass in bank voles, wood mice, and common shrews captured in South-East Norway (2018–2022). Shaded areas denote respective 95% confidence intervals, points denote raw data observations, and dashed lines denote mean body mass per species

Average larval tick infestations were highest on the wood mouse (39.4 larvae/host), followed by the bank vole (26.4 larvae/host) and common shrew (24.0 larvae/host; Fig. 2a). The best supported larval tick infestation model included species, sex, and species-centred (log) body mass, and the interaction between “sex × species-centred (log) body mass”, while season and interactions between “species × sex” “species × species-centred (log) body mass” were not included in the most parsimonious model (Table S7). Larval tick infestation was higher in males and increased with body mass for all species. The increase in larval tick infestation with log-body mass was stronger for males than females (Fig. 4a-c; Table 1). When body mass was not included, there was a significant effect of season, with 1.4 [1.2–1.7] times higher larval infestations in spring than in fall (*P* = < 0.001).

The best supported model of nymphal tick presence included species, sex, and species-centred (log) body mass, and the interaction between “sex × species-centred (log) body mass”, while season and interactions between “species × sex” “species × species-centred (log) body mass” were not included in the most parsimonious model (Table S7). Average nymphal tick intensity was highest in wood mice (4.1 nymphs/host), followed by the bank vole (3.0 nymphs/host) and common shrew (1.5 nymphs/host; Fig. 2b). The best supported model of nymphal tick intensity (for bank voles and wood mice only) included the same parameters as for nymphal tick presence except species (Table S8). Nymphal tick presence and intensity increased with body mass, and it increased more for males than for females (Fig. 4d–f; Fig. S2; Table 1). There was no significant effect of season on nymphal tick presence even when body mass was replaced with season.

The best supported model of *B. burgdorferi* s.l. infection included species, sex, species-centred (log) body mass, squared species-centred (log) body mass, in addition to interactions between “species × sex”, “sex × species-centred (log) body mass”, and “species × species-centred (log) body mass” (Table S7), while season was not included in the most parsimonious model. Prevalence of *B. burgdorferi* s.l. increased with body mass for both the bank vole and wood mouse with a greater increase for males than for females (Table 1). Relative to the mean (log) body mass in respective species, infection probability increased with body mass for bank voles and wood mice (Fig. 4g–h). For the bank vole, the relationship flattened out for heavy males and females (Fig. 4g). For the common shrew, infection was female biased (Table 1). Specifically, female shrews of low to average body mass were more likely to be infected than males of equivalent body mass, but with wide confidence bands indicating considerable variation in this pattern (Fig. 4i). For the bank vole and wood mouse, adding nymphal tick presence to the above model explained further variation in infection prevalence (Table S8), whereby both males and females parasitised by nymphs were more likely to be infected with *B. burgdorferi* (Fig. S3; Table 1). There was no significant effect of season on *B. burgdorferi* s.l. infection even when body mass was replaced with season.

## Discussion

In most host-parasite systems, individual heterogeneity in hosts is an important driver of parasite distribution, and heavily infested individuals contribute disproportionate numbers of parasites into the environment and host population (Wilson et al. 2002). Ixodid tick densities at the individual host level, both between and within species, determines individual and species level heterogeneity in pathogen infection, which has major implications for the circulation of zoonotic pathogens in ecosystems (Brunner and Ostfeld 2008; Kilpatrick et al. 2017). In our study, we focussed on patterns of tick infestation by *I. ricinus* and infection prevalence of *B. burgdorferi* s.l. in small mammals depending on host species, sex, and body mass. Ixodid larvae were found on virtually all individual hosts of all species except pygmy shrews. We found that wood mice maintained the highest tick infestation but were less often infected with *B. burgdorferi* s.l., while common shrews and bank voles maintained lower tick infestations than wood mice but were more often infected with *B. burgdorferi* s.l.

The feeding of both nymphal and larval *I. ricinus* on small mammals is important for the circulation of *B. burgdorferi* s.l. Nymphal ticks carrying *B. burgdorferi* s.l. establish infection in their host during blood feeding so the pathogen can replicate and infect larvae subsequently feeding on the same host (van Duijvendijk et al. 2016; Kahl and Gray 2023). We found that both larval and nymphal tick infestations were positively correlated with body mass across all three host species as hypothesised (IA). Similar findings have previously been reported in several studies of tick burden on small mammals (Harrison et al. 2010; Mysterud et al. 2015; Perez et al. 2017; Ferrari 2022; Perez 2022) as well as for other ectoparasites (Surkova et al. 2018). Higher load likely results from larger individuals providing a larger surface area for more ticks to attach to (Arneberg et al. 1998). Body mass is also linked to home range size (Harestad and Bunnell 1979; Godsall et al. 2014), which may affect space use-mediated exposure risk to questing ticks (Dallas et al. 2012; Devevey and Brisson 2012). We found that infection prevalence also increased with body mass in bank voles and wood mice in accordance with our hypothesis (IB). This is similarly consistent with previous studies on bank voles and wood mice in France (Perez et al. 2017) as well as bank voles in Sweden (Tälleklint et al. 1993). The body mass of bank voles and common shrews were higher in spring than in fall, which likely reflects an influx of small offspring between spring and fall. This was also reflected in larval tick infestations where tick infestations were higher in spring than in fall, but not after body mass was included. Hence, demographic shifts may drive overall tick infestation levels from predominantly adults with higher tick infestations in spring towards younger and lighter individuals with lower tick infestations in fall. Furthermore, nymph presence was positively correlated with *B. burgdorferi* s.l. infection probability in the bank vole and wood mouse beyond the effect operating through host sex and body size (Fig. S2), supporting the idea that nymphs are directly involved in establishing the infection in hosts (van Duijvendijk et al. 2016). Evidence from our study suggests that large individuals, presumed older, contribute proportionally more to maintaining tick infestations across three different species of small mammals, and that large bank voles and wood mice are more likely to be infected with *B. burgdorferi* s.l. than smaller individuals.

A commonly reported pattern in mammals is a higher level of parasitism in males than females (Zuk and McKean 1996; Schalk and Forbes 1997; Krasnov et al. 2012). Similar to previous studies, we found that males had higher tick infestations than females (Fig. 4a–f; Fig. S1). However, this did not correspond to sexual body mass dimorphism, because males were not larger than females in any of the three species even when pregnant females were accounted for. Male-biased parasitism but no sexual-body mass dimorphism was similarly evident in bank voles in France (Perez 2022) indicating that male-biased parasitism is linked to other factors. An alternative explanation for male-biased parasitism is sex-differences in exposure. In small mammals, home ranges are often larger in males compared to females (Godsall et al. 2014), including in our three main study species (Shillito 1963; Attuquayefio et al. 1986; Boratyński et al. 2020). Thus, increased activity of mature males, even if they are not larger in size than adult females, could increase their exposure risk to questing ticks.

Pathogens are thought to have a narrower host niche than their tick vectors (Estrada-Peña et al. 2016; Estrada-Peña and Fuente 2017). Patterns of infection prevalence might thus differ from patterns of tick aggregation across taxa. The focal species of both rodents and shrews in our study are known as competent hosts to both *I. ricinus* and *B. afzelii* (Mysterud et al. 2019a), but several aspects of individual heterogeneity remain unclear. In a laboratory study on house mice (*Mus musculus*), males and females were exposed to *I. scapularis* nymphs infected with *B. burgdorferi* sensu stricto (Zinck et al. 2022), the respective vector and genospecies most often involved in Lyme disease transmission in North America (Piesman and Gern 2004). Even at the same level of exposure, males had on average 1.45 times higher spirochete abundance in tissues compared to females, indicating a sex-difference in immune response to the bacteria (Zinck et al. 2022). Indeed, sex biases in parasitism and infection may reflect hormone mediated trade-offs in life-history traits, whereby mature males often invest more energy into mating effort at the expense of parasite resistance and immune response (Hughes and Randolph 2001; Mills et al. 2010; Córdoba-Aguilar and Munguía-Steyer 2013; Henttonen 2022). Moreover, a male-biased shift in infection prevalence with increasing body mass, as evident in bank voles and wood mice (Fig. 4g–h), could thus be linked to sexual maturation and elevated levels of immunosuppressive testosterone in males (Trigunaite et al. 2015; Henttonen 2022).

Most studies of tick-borne pathogens in small mammals only concern rodents. Common shrews are important hosts for feeding instar stages of ixodid ticks (Mysterud et al. 2015; De Pelsmaeker et al. 2022), and they can maintain significant reservoirs for tick-borne pathogens in some ecosystems (Bown et al. 2011; Mysterud et al. 2019b; Sormunen et al. 2023). Shrews differ in life history strategy compared to rodents by being shorter lived and exerting much higher metabolic rates (Gliwicz and Taylor 2002). There was no increase in infection with higher body mass in shrews, and infection was female-biased rather than a male-biased (Fig. 4i; Table 1). The lack of an increase with body mass could potentially imply that *B. burgdorferi* s.l. infections are cleared in overwintered shrews, in contrast to rodents that are known to retain the infection though winter (Humair et al. 1999), or that the shorter life span of shrews leads to a higher turn-over of individuals. *Sorex* shrews show extraordinarily high reproductive effort and their offspring are born highly altricial in large litters (Gittleman and Thompson 1988; Genoud and Vogel 1990), which could imply trade-offs in reproduction at the expense of infectious disease resistance (Buckingham et al. 2023). This could further imply that mainly uninfected shrews grow to higher body mass. The common shrew provides an interesting contrast to rodents, with a different life history and trade-offs, highlighting how patterns of individual heterogeneity may differ between species from different taxonomic groups.

Our study highlights how patterns of individual heterogeneity in parasitism can differ among taxonomic groups of small mammals, and that parasite infestation levels as well as the likelihood of pathogen infection can be male-biased independently of sexual body size-dimorphism. The focal pathogen (*B. burgdorferi* s.l.), parasite (*I. ricinus*), and small mammalian hosts form core parts of the transmission cycle of Lyme disease at northern latitudes of Europe, and an improved understanding of the role of hosts ultimately have relevance for human health (Estrada-Peña and Fernández-Ruiz 2023).

### Supplementary Information

Below is the link to the electronic supplementary material.

Supplementary file1 (PDF 367 KB)

## Data Availability

Data and scripts are available at Zenodo: 10.5281/zenodo.10102122.
