# Supplementary material for: Individual heterogeneity in ixodid tick infestation and prevalence of Borrelia burgdorferi sensu lato in a northern community of small mammalian hosts
Source: Oecologia. 2023 Nov 13;203(3-4):421–33. doi: 10.1007/s00442-023-05476-w (PMC10684702; doi:10.1007/s00442-023-05476-w)
Supplement: Supplementary file 1 — Supplementary file1 (PDF 367 KB) [file 442_2023_5476_MOESM1_ESM.pdf]

Online Resource 1 - Appendices

Individual heterogeneity in ixodid tick infestation and prevalence of *Borrelia burgdorferi* sensu lato in a northern community of small mammalian hosts

Lars K. Lindsø, Jason L. Anders, Hildegunn Viljugrein, Anders Herland, Vetle M. Stigum, W. Ryan Easterday, and Atle Mysterud

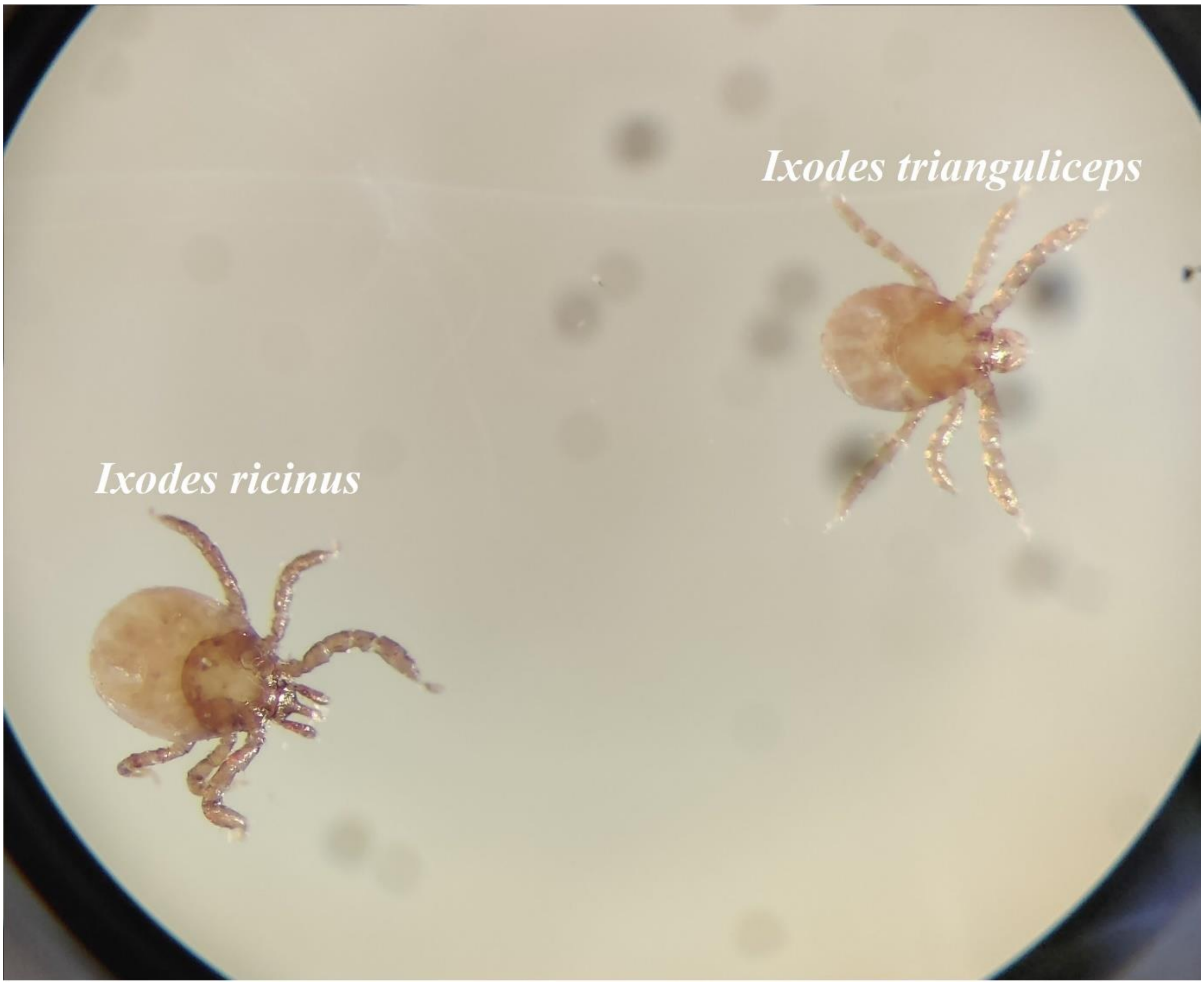

Figure S1: Exemplification of morphological difference in *Ixodes ricinus* (left) and *Ixodes trianguliceps* (right) larvae under a stereomicroscope. Note the palpi on each side of the hypostome, with long, parallel palpi with basal narrowing in *I. ricinus* versus the short, broad, and convex palpi of *I. trianguliceps* (Arthur 1963). Photo: Lars Lindsø.

Table S1: Sequences and adjusted primer set and probe concentrations in respective multiplex real-time quantitative PCR assays for detecting *B. burgdorferi* s.l. and *A. phagocytophilum* and *I. ricinus* and *I. trianguliceps*, with respective target genes, product size (bp), sequences, and concentration (µM). Infection prevalence of *A. phagocytophilum* was not considered in the present study.

Infection prevalence	**Target**	**bp**	**Sequence**	**µM**
A. phagocytophilum	16S rRNA	75		
F primer			ATGGAAGGTAGTGTTGGTTATGGTATT	0.900
R primer			TTGGTCTTGAAGCGCTCGTA	0.900
Probe			[HEX]TGGTGCCAGGGTTGAGCTTGAGATTG[TAMRA]	0.125
B. burgdorferi s.l.	23S rRNA	77		
F primer			CGAGTCTTAAAAGGGCGATTTAGT	0.700
R primer			GCTTCAGCCTGGCCATAAATAG	0.700
Probe			[6FAM]AGATGTGGTAGACCCGAAGCCGAGTG[TAMRA]	0.175
Tick species				
I. ricinus	*ITS2*	72		
F primer			TTGACTGTGTCGGATCGTGG	0.600
R primer			TCGCTCCGAGAACGTGAAAA	0.600
Probe			[5TexRd-XN]ACGCATGTTTATGCTTTCTTGCGTTGCG[Iowa black]	0.200
I. trianguliceps	*ITS2*	128		
F primer			CGCGAACGTTGGAATCGTAC	0.600
R primer			GGCTCCAGTATTCATCGGGG	0.600
Probe			[5HEX]CCGTGCGTCGTAGCCATCCGT[3IAbRQSp]	0.200

Table S2: An overview of samples sizes of captured small mammals per season between 2018 and 2022 in Son, Viken county, Norway.

	**2018**		**2019**		**2020**		**2021**		**2022**		
	Spring	Fall	Spring	Fall	Spring	Fall	Spring	Fall	Spring	Fall	Sum
A. sylvaticus	2	9	20	16	27	30	23	11	11	7	156
Microtus agrestis	1	0	0	3	0	9	0	0	0	1	14
M. glareolus	3	16	4	12	10	143	10	27	10	23	258
S. araneus	2	27	1	21	1	36	1	14	0	3	106
S. minutus	0	3	0	5	3	11	0	1	0	0	23
Sum	8	55	25	57	41	229	34	53	21	34	557

Table S3: Overview of individual larval and nymphal ticks from captured small mammal hosts in Son, Viken county, Norway between 2018-2022, identified by species using a multiplex real-time quantitative PCR assay (2018-2019) and by morphology (2020-2022).

	**2018**	**2019**	**2020**	**2021**	**2022**	**Total**
Method	qPCR	qPCR	Morphology	Morphology	Morphology	
Larvae						
% *I. ricinus*	98.9	99.6	98.5	99.5	99.9	99.1
n *I. ricinus*	1020	466	5646	3234	2063	12429
n *I. trianguliceps*	11	2	87	16	3	119
Total	1031	468	5733	3250	2066	12548
Nymphs						
% *I. ricinus*	94.1	98.6	91.0	96.2	98.6	95.2
n *I. ricinus*	16	70	183	153	139	561
n *I. trianguliceps*	1	1	18	6	2	28
Total	17	71	201	159	141	589

Table S4: An overview of sample sizes of each tick species from captured small mammalian hosts between 2018 and 2022 in Son, Viken county, Norway. The table shows the number of individuals of larval and nymphal ticks per species, the total number of ticks including unidentified ticks, the percentage of *Ixodes ricinus* of all species identified ticks (% *I. ricinus*), and percentage of species-determined ticks (% SDT) by morphology and qPCR per small mammal species.

Larvae	**Sum *I. ricinus***	**Sum *I. trianguliceps***	**Total**	**% *I. ricinus***	**% SDT**
A. sylvaticus	4438	11	6142	99.75	0.72
Microtus agrestis	251	2	265	99.21	0.95
M. glareolus	5885	83	6783	98.61	0.88
S. araneus	1727	14	2449	99.20	0.71
S. minutus	128	9	168	93.43	0.82
Sum	12429	119	15807	99.05	0.79
Nymphs					
A. sylvaticus	295	7	329	97.68	0.92
Microtus agrestis	39	0	39	100.00	1.00
M. glareolus	208	18	253	92.04	0.89
S. araneus	19	3	24	86.36	0.92
S. minutus	0	0	0	NA	NA
Sum	561	28	645	95.25	0.91

Table S5: Results of model selection of body mass in bank voles, wood mice, and common shrews captured in South-East Norway (2018-2022) as a function of season, sex, species, and their interactions. Gravid females were excluded from the analysis. The table shows the parameters included in the top five models (+), number of parameters (df), AIC, and model weight. The model used for inference is highlighted in grey.

Intercept	season	sex	species	season:sex	season:species	sex:species	df	AICc	ΔAIC	Weight
**2.854**	+		+		+		**9**	**2592.3**	**0**	**0.40**
2.886	+	+	+		+	+	12	2593.3	0.95	0.25
2.864	+	+	+		+		10	2594.0	1.67	0.17
2.87	+	+	+	+	+		11	2595.3	2.98	0.09
2.885	+	+	+	+	+	+	13	2595.3	3.03	0.09

Table S6: Parameter estimates from the generalized linear mixed models of larval *Ixodes* tick intensity and presence of *Ixodes* nymphs on small mammals captured in South-East Norway (2018-2022) as a function of whether the host was found alive or dead (live/dead), log-transformed body mass, and their interaction.

Parameter	Estimate	Std. error	*z*	*P*
Larval tick intensity	(log-link)			
Intercept [1]	1.679	0.289	5.817	< 0.001
(log) body mass	0.595	0.094	6.339	< 0.001
live/dead = live	0.226	0.439	0.515	0.606
(log) body mass: live/dead = live	-0.013	0.157	-0.080	0.936
Nymphal tick presence	(logit-link)			
Intercept [1]	-5.723	0.917	-6.243	< 0.001
(log) body mass	1.875	0.324	5.782	< 0.001
live/dead = live	0.596	1.542	0.386	0.699
(log) body mass: live/dead = live	-0.110	0.534	-0.206	0.837

[1]: Corresponds to an individual of body mass = 1 g captured and found dead.

Table S7: Model selection results of A) number of *Ixodes* larvae, B) presence of *Ixodes* nymphs, and C) prevalence of *B. burgdorferi* s.l. in small mammals captured in South-East Norway (2018-2022). The table shows the parameters included in the top five models (+), number of parameters (df), AIC, and model weight. Body mass (mass) was log-transformed and species-centred. Models used for inference are highlighted in grey.

Intercept	mass	season	sex	species	mass:sex	mass:species	sex:species	df	AICc	ΔAIC	Weight
A) Larval tick burden											
3.162	+	+	+	+	+			10	4276.9	0	0.24
3.162	+	+	+	+	+	+		12	4277.0	0.15	0.22
3.182	+		+	+	+	+		11	4277.0	0.16	0.22
**3.186**	**+**		**+**	**+**	**+**			**9**	**4277.8**	**0.90**	**0.15**
3.152	+	+	+	+	+		+	12	4280.1	3.25	0.05
B) Nymphal tick presence											
-0.579	+	+	+	+	+			9	558.6	0	0.40
**-0.667**	**+**		**+**	**+**	**+**			**8**	**559.3**	**0.69**	**0.28**
-0.564	+	+	+	+	+		+	11	561.8	3.19	0.08
-0.607	+	+	+	+	+	+		11	562.1	3.46	0.07
-0.653	+		+	+	+		+	10	562.5	3.88	0.06
C) Infection prevalence											
-0.375	+	+	+	+	+	+	+	11	554.4	0	0.392
**-0.466**	**+**		**+**	**+**	**+**	**+**	**+**	**10**	**555.2**	**0.72**	**0.274**
-0.071	+	+	+	+	+	+		13	555.7	1.3	0.205
-0.165	+		+	+	+	+		12	556.8	2.34	0.122
-0.551	+	+	+	+		+	+	12	563.9	9.5	0.003

Table S8: Results of model selection of A) *Ixodes* nymph intensity, and B) prevalence of *B. burgdorferi* s.l. in bank vole (*M. glareolus*) and wood mouse (*A. sylvaticus*) captured in South-East Norway (2018-2022). The table shows the parameters included in the top five models (+), number of parameters (df), AIC, and model weight. Body mass (mass) was log-transformed and species-centred. Models used for inference are highlighted in grey.

Intercept	mass	nymph	season	sex	sp	mass:sex	mass:sp	sex:sp	df	AICc	ΔAIC	Weight
A) Nymphal tick burden												
-0.391	+			+	+	+		+	9	644.1	0	0.24
**0.066**	**+**			**+**		**+**			**7**	**645.0**	**0.89**	**0.15**
-0.079	+			+	+	+			8	645.6	1.47	0.12
-0.050	+		+	+		+			8	645.9	1.82	0.10
-0.405	+		+	+	+	+		+	10	646.2	2.13	0.08
B) Infection prevalence												
-0.627	+	+	+	+	+	+			9	400.9	0	0.24
**-0.739**	**+**	**+**		**+**	**+**	**+**			**8**	**401.3**	**0.39**	**0.20**
-0.690	+	+	+	+	+	+		+	10	402.3	1.48	0.11
-0.617	+	+	+	+	+	+	+		10	402.6	1.78	0.10
-0.800	+	+		+	+	+		+	9	402.8	1.90	0.09

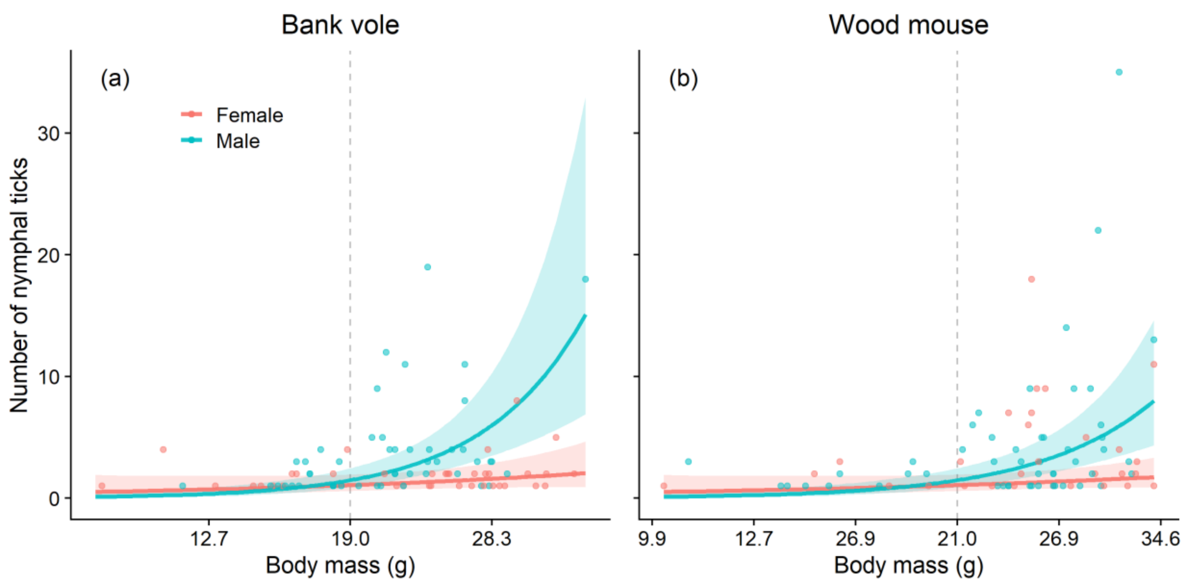

Figure S2: Predicted *Ixodes* nymph intensity as a function of sex and log-transformed body mass centred by species in (a) bank vole (*M. glareolus*) and (b) wood mouse (*A. sylvaticus*) captured in South-East Norway (2018-2022). The x-axis is on log-scale, and x-axis labels denote back-transformed values of body mass (g). Shaded areas denote respective 95% confidence intervals, points denote raw data observations, and dashed lines denote mean body mass.

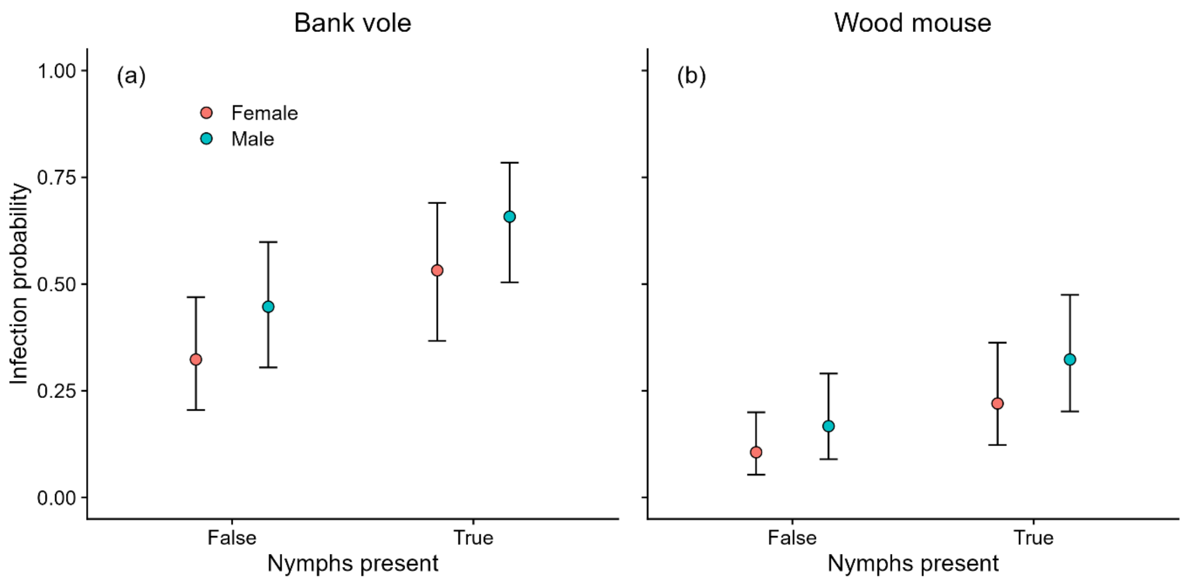

Figure S3: Predicted prevalence of *B. burgdorferi* s.l. as a function of sex and if nymphs were found on the host in (a) bank vole (*M. glareolus*) and (b) wood mouse (*A. sylvaticus*) captured in South-East Norway (2018-2022). Predicted values are shown for body mass = mean log-transformed body mass of each species. Error bars denote respective 95% confidence intervals.
